# Attitudes towards and experiences with economic incentives for engagement in HIV care and treatment: Qualitative insights from a randomized trial in Kenya

**DOI:** 10.1371/journal.pgph.0000204

**Published:** 2022-02-23

**Authors:** Sarah Iguna, Monica Getahun, Jayne Lewis-Kulzer, Gladys Odhiambo, Fridah Adhiambo, Lina Montoya, Maya L. Petersen, Elizabeth Bukusi, Thomas Odeny, Elvin Geng, Carol S. Camlin

**Affiliations:** 1 Centre for Microbiology Research, Kenya Medical Research Institute, Kisumu, Kenya; 2 Department of Obstetrics, Gynecology & Reproductive Sciences, University of California, San Francisco, California, United States of America; 3 Division of Biostatistics, School of Public Health, University of California Berkeley, Berkeley, California, United States of America; 4 Divisions of Biostatistics and Epidemiology, School of Public Health, University of California Berkeley, Berkeley, California, United States of America; 5 Department of Medicine, University of Missouri-Kansas City, Kansas City, Missouri, United States of America; 6 Division of Infectious Diseases, Department of Internal Medicine, Washington University, St. Louis, Missouri, United States of America; 7 Division of Prevention Science, Department of Medicine, University of California, San Francisco, California, United States of America; University of Bristol, UNITED KINGDOM

## Abstract

Growing literature has shown heterogenous effects of conditional cash incentives (CCIs) on HIV care retention. The field lacks insights into reasons why incentives impact various patients in different ways–differences that may be due to variations in psychological and social mechanisms of effect. A deeper understanding of patients’ perceptions and experiences of CCIs for retention may help to clarify these mechanisms. We conducted a qualitative study embedded in the ADAPT-R trial (NCT#02338739), a sequential multiple assignment randomized trial (SMART) that evaluated economic incentives to support retention in HIV care among persons living with HIV (PLHIV) initiating antiretroviral therapy in Kenya. Participants who attended their scheduled clinic visits received an incentive of approximately $4 each visit. Interviews were conducted between July 2016 and June 2017 with 39 participants to explore attitudes and experiences with economic incentives conditional on care engagement. Analyses revealed that incentives helped PLHIV prioritize care-seeking by alleviating transport barriers and food insecurity: “*I decided to forgo [work] and attend clinic […] the voucher relieved me*”. Patients who borrowed money for care-seeking reported feeling relieved from the burden of indebtedness to others: “*I borrow with confidence that I will pay after my appointment*.” Incentives fostered their autonomy, and enabled them to support others: “*I used the money to buy some clothes and Pampers for the children*.” Participants who were intrinsically motivated to engage in care (“*my life depends on the drugs*, *not the incentive*”), and those who mistrusted researchers, reported being less prompted by the incentive itself. For patients not already prioritizing care-seeking, incentives facilitated care engagement through alleviating transport costs, indebtedness and food insecurity, and also supported social role fulfillment. Conditional cash incentives may be an important cue to action to improve progression through the HIV treatment cascade, and contribute to better care retention.

## Introduction

Despite the tremendous gains in the global HIV response, suboptimal HIV care retention continues to place people living with HIV (PLHIV) at risk of poor health outcomes, undermining progress towards elimination [1–5]. Lifetime antiretroviral adherence is challenging: in sub-Saharan Africa (SSA), distance to clinics and associated transport and time costs, stigma and denial, and health system issues such as crowded facilities and long wait times, all contribute to care interruption [6–10]. These challenges result in nearly one-third of patients in HIV care becoming lost-to-follow up after 24 months, with increases in attrition over time [3,11,12].

Economic incentives hold promise for addressing the persistent challenges to HIV care engagement. Economic transfers are direct or indirect regular and predictable non-contributory payments that raise income with the objective of reducing poverty and vulnerability; they may by conditional or unconditional [13]. Prior research has shown the potential of economic incentives to improve treatment adherence and reduce loss-to-follow up among PLHIV [14,15], and documented the behavioral effects of incentives on HIV prevention, medication ownership, treatment retention, and HIV testing uptake [16–25]. Incentives can ‘nudge’ individuals toward adopting a healthy behavior by increasing immediate benefits to promote HIV testing or HIV care linkage [26,27], and may serve as a useful addition to behavioral change toolkits [28]. In low-resource settings, incentives may also provide the resources to mitigate structural and economic constraints such as costs of transportation to clinics [19,24,29].

Despite these positive results, other studies have failed to show evidence for positive effects of economic incentives on HIV outcomes. A recent review of economic incentives trials showed that numerous US-based studies showed that incentives improved linkage to care but resulted in no difference in virologic suppression, and limited durability beyond the incentive period [30]. A study in Uganda found similar results, with monetary incentives having no effects on viral load suppression; however, high viral load suppression among the cohort at baseline may have played a role [31]. Others have also shown that although economic incentives tend to improve care engagement in the short-term, their role in improving long-term care engagement remains uncertain [14,25,30,32].

The mixed evidence base highlights the need for in-depth qualitative studies to elucidate the pathways by which economic incentives affect retention outcomes, both among patients who achieve positive health outcomes and among those that do not. Understanding what incentives mean to patients who receive them may help explain why incentives work in some settings and patients but not in others. Findings may thus help to inform development of effective behavioral incentive interventions to improve HIV care outcomes. We therefore conducted a qualitative sub-study nested within a larger randomized controlled trial of conditional cash transfers to support retention in HIV care in Kenya. We investigated the perceived effects of receiving incentives on patients’ experiences of care, and barriers and motivators for care-seeking decisions, in order to better understand the role of economic incentives in helping patients overcome barriers to care.

## Methods

### Study population and settings

We conducted a qualitative study embedded in the ADAPT-R Trial (Adaptive Strategy for Preventing and Treating Lapses of Retention in HIV Care -NCT#02338739), a trial to evaluate strategies focused on optimizing HIV retention and health in the Nyanza region of western Kenya. Patients were eligible for enrollment in the qualitative study if they were adult (aged >18 years) PLHIV initiating ART at an HIV clinic (Lumumba and Pandi were urban sites, Ahero and Rongo were rural sites, and Migori a peri-urban site), if they were among the n = 658 patients randomly assigned to receive a small cash incentive, and if they had not missed their first clinic visit, to ensure that they had experience receiving the incentive following enrollment into the study. We used parent trial data to select a purposive sample of participants balanced by broad age, gender and clinic groupings, at a defined recruitment time period. During that period, n = 39 were identified as eligible and selected to participate in the interviews. The sample of 39 participants were approximately balanced by gender (n = 18 male, n = 21 female), clinic type (Lumumba = 9, Migori = 11, Rongo = 9, Ahero = 9 and Pandi = 1), and age (aged 19 to 54 years, median age 29). Sample selection was stopped after recruitment of the n = 39 to align with study timeline requirements.

Patients in the cash incentive arm who did not miss their clinic visits and attended their clinic visits within three days of their scheduled clinic date, received an incentive of $4. The incentive was dispensed at the clinic by the study staff on completion of the clinic visit. After visiting the pharmacy and scheduling their next clinic visit, study participants obtained their cash at a designated study desk. Participants signed a receipt of incentive payment which the study kept for accountability and documentation.

### Data collection

The study team was led by an investigator with expertise in qualitative research (CC); a female Kenyan qualitative researcher highly trained in qualitative research methods and fluent in Dholuo and Kiswahili, (GO), conducted the interviews, which were audio-recorded, transcribed and translated into English. The participants were newly enrolled into the main study, and therefore had no prior interviewer-participant relationship.

A list of participants eligible for interview was generated by the qualitative researcher. Study research assistants provided the qualitative researcher with participants demographics and phone numbers. The qualitative researcher called, introduced herself and reason for calling and scheduled appointments with them. The interviews were conducted at the clinic in a private room with only the interviewer and the participant ensuring confidentiality and privacy. Once the participants were in the clinic they were taken through the consenting process (ensuring participant language preference) by the qualitative researcher and upon consenting proceeded with the in-depth interview. None refused to participate or dropped out. The participants were reimbursed for their time and transport. Interviews were conducted from July 2016 to June 2017, within a month of study enrollment, following patients’ clinic visits and counseling for ART. Data were collected following diagnosis and during the early stages of HIV-care linkage. Semi-structured interview guides explored participants’ perceptions, attitudes, and preferences related to economic incentives, as well as their risk-taking, risk aversion, partner and other social support for care-seeking, and other psychosocial factors influencing care engagement (S1 Table shows selected topics and probes). All interviews were conducted in participants’ preferred language (English, Dholuo or Swahili), audio recorded and were 60–90 minutes in length.

### Data analysis

A five-person female qualitative team (SI,GO,FA,JK,MG) and one male (EG), including the data collector and Kenyan study team who were native speakers of the local languages, coded transcripts and participated with the project lead (CC) in the analysis and interpretation of data. Audio recordings were translated, transcribed into English. Transcripts were then reviewed and stored in a password protected folder and backed up on the cloud. Coding was done inductively and deductively, using a collaboratively developed and theory-informed coding framework based on the domains of inquiry in the interview guides. The coding framework was iteratively refined during the coding process based on empirical data and discussions with the full team. Dedoose software was used for coding. Codes were queried, coded segment within thematic categories were extracted and revised, and analyzed using thematic analysis, in the domain of interpretivist approaches in qualitative research [33,34]. Further, analysis and interpretation were done by the full study team, including data collectors, strengthening our analytical approach and rigor.

### Ethics approvals

All participants provided written informed consent to participate. The study was approved by the institutional review boards at the Kenya Medical Research Institute (KEMRI) (SSC No 2838) and the University of California, San Francisco (CHR-13-12810).

## Results

In the results that follow, we present both participants’ direct attributions for their care-seeking or care-avoidant behaviors, and also our assessment of evidence for the factors influencing their behaviors through our interpretation of interview narratives. Although many patient interview narratives suggested that conditional cash transfers influenced patient care decisions, others showed limited or no evidence of influence of incentives on care seeking decisions. We first discuss participant perceptions of positive influences of CCIs on care decisions, and then highlight narratives in which participants reported incentives to have limited influence. (To protect confidentiality, only participants’ gender, age and clinic location are shown for each excerpt).

### Alleviating transport barriers and livelihood challenges

Narratives revealed that livelihood-related opportunities sometimes directly conflicted with patients’ HIV care-seeking intentions. In these instances, incentives helped to resolve this tension. Patients reported that the incentives helped them to have more control over their schedule, enabling them to better plan and balance care-seeking with livelihood strategies and to prioritize care. In particular, patients reported using incentives to cover transport costs, which in turn reduced time spent traveling to and from clinics among patients who would otherwise walk long distances.

“*I felt good because [the incentive] encourages people especially on the money matters*, *it encourages people for transport especially those who are coming from far*, *not around this [clinic] place*. *They can be assured that when they come here*, *they can get something*, *transport*, *to go back with*.”

Male, 46, Pandi

This in turn also reduced the stress related to the cost burden of attending clinic. In the examples below, a man and a woman discuss how not worrying about the costs of transport reduced the perceived psychological burden of living with HIV:

“*[The incentive] has covered my transport costs*. *It makes me come to the hospital with ease*. *I do not worry about transport and this reduces the burden of living with HIV*.*”*

Female, 27, Ahero

“*You know*, *you cannot be sad when you get transport because you will be going at no loss if you come to the clinic (laughs) you will not spend your money*.*”*

Male 30, Migori

A woman below discusses how receiving an incentive allowed her to arrive at the clinic on time, and return to her home early:

“*[The incentive] enables me to come to the clinic in good time for my medication then go back home early*.*”*

Female 24, Rongo

Similarly, a man below discussed how he was able to have guaranteed transportation, and as a result, felt reduced fatigue and worry:

“*Because I’m sure that I will have an effective means of getting back home; I won’t reach home a tired person because I can use it [incentive] to board a motorbike*.*”*

Male, 24, Lumumba

Yet, incentives were not always used to pay for transport. Narratives suggested that incentives also worked to expand participants’ agency or the perceived set of day-to-day options and choices for how to spend their time, money and effort. This expansion of agency or choice of options worked to support care seeking. A man below discusses how he used the money to fix his clock radio, which he used as a reminder to take his medication:

“*You know [incentive] is money*, *so I planned on how I would use it*, *I had told you that my radio had broken so I would plan to go and use the cash to repair it (laughs) so that it can help me know about my time for medication*.*”*

Male, 30, Migori

Narratives reveal how participants had more energy and time to pursue other activities because of the expanded day-to-day life choices that the incentives offered:

“*I took long [to travel to clinic] but I left with something; in that case even if you board a motorbike to go back home*, *you won’t have the worries of going to work*. *You will just get back home*, *sit home*, *relax and take your medication as you wait for your sleeping time […]that’s something that motivates me to come because when I come [to the clinic]*, *I will not go to my work which is just a salon and most times I just rest*…”

Female, 23, Ahero

“*I thanked God*, *the day had not gone to a waste since in a day I make a profit of Ksh 600*, *but that day I decided to forgo that and attend clinic*. *Getting the [incentive] relieved me*. *That day I decided to wake up and go the clinic first and then I would later attend to my businesses*.”

Male, 30, Lumumba

The incentives also elicited anticipation and positive emotions related to care-seeking. Participants’ increased motivation to attend clinic, and their heightened attentiveness to the clinic visit date are illustrated in the excerpts below:

“*I would think of the incentive; I would just feel like coming to the clinic*.*”*

Female, 32, Ahero

“*[The incentive] is good*. *It keeps lingering in my mind and I can’t wait for the next clinic visit to get the [incentive]*. *I am not worried about transport because I know that I will get it when I come*. *It is motivating me to come*.*”*

Female, 48, Migori

### Reducing financial insecurity and indebtedness

A second key theme concerned ways in which economic incentives relieved participants of indebtedness to others, allowing them to avoid borrowing money to pay for clinic transportation. This relieved anxiety and helped patients to manage social and interpersonal pressures. Participants discussed how being able to fulfill outstanding debts, or comfortably borrow money that they knew they could repay, reduced the anxiety they used to feel when they borrowed to attend clinic:

“*You know when you want to come to the clinic and you do not have transport*, *you will be forced to work for someone on the farm or even borrow the money which you may have difficulty in paying back*. *So [incentive] is very good […] when I do not have transport money*, *I just borrow with confidence that I will pay after the clinic appointment*.”

Female, 37, Rongo

“*I do not have a job at the moment and so if you continue giving me the [incentive]*, *I can use 100 shillings for fuel to come to the clinic and go back home; even if I borrow some money from someone*, *I can use the proceeds from the [incentive] to refund the lender*…”

Male, 54, Migori

“*This [incentive] motivates me to come since sometimes you can be broke and then borrow from someone then when you get the incentive you can pay back*. *It can motivate someone to come*.”

Male, 30, Migori

Finally, a participant discusses how the incentive allows him to no longer be indebted to others.

“*It really helped me*, *I saved it on Mpesa (cash stored on phone) and withdrew it on the day I was visiting the clinic to use it on transport*. *Now*, *I can lack any other thing but not transport*. *Before*, *I would be broke*, *go borrow some 300 shillings from a friend to be refunded later though I was not sure where I would get the 300 to refund*. *In fact*, *I really appreciate*, *even when you called me*, *I told you that I was coming*.”

Male, 30, Lumumba

### Reducing food insecurity

A third key identified theme concerned ways in which economic incentives alleviated food insecurity among participants living in economically disadvantaged households. This in turn facilitated care-seeking by relieving participants of the difficult trade-off between using limited money for food purchases versus clinic visits. The ways in which participants used incentives were flexible, with each patient deciding and reporting on various uses. Our data reveal that in addition to transport, the incentive was important for obtaining and replenishing household food supplies:

“*It also helps me in buying some household items before I get my salary; my husband had an accident with his motorcycle so he’s not able to work for now*. *I am able to buy things like vegetables*, *water and so on*.”

Female, 20, Lumumba

The men below discuss how they purchased food, and alleviated their hunger:

“*I am no longer worried when I spend a lot of time at the clinic because the money that remains after using it for transport helps me in buying food for dinner*”

Male, 25, Rongo

“*The money*, *therefore*, *enabled me to purchase some of the items I lacked*. *I would also be hungry and with no transport*, *I had to not only endure hunger but also walk to the hospital*.”

Male, 20, Migori

The ability to provide for others, in contrast to being indebted to others, also facilitated participants’ social role fulfillment, particularly roles as parents and spouses. It helped pay for food which participants reported sharing with their children or family:

“W*hen I leave the place*, *I leave with some money such that even if I lack food for supper*, *I will be certain that my child will eat together with me*.”

Female, 23, Ahero

In addition to helping buy food for families, the incentive was reported to have provided variety in the types of food purchased:

“*My wife usually doesn’t take meat; on that day I took Managu (traditional vegetable) and some of the fruits and the bread of course*. *I was not aware that I could get that amount of money; I was happy my family was also happy*.”

Male, 43, Migori

Participants expressed feeling content upon receipt of the incentive and buying food, as demonstrated by the participants below:

“*I felt good because I bought for myself some good lunch (both laugh)*. *I did -I got myself lunch of course*!”

Male, 44, Lumumba

“*Receiving money for transport and having been hungry the whole day plus I didn’t open my business the whole day; it was worth being happy for*! *I was very happy and for a moment*, *I forgot that I was HIV positive*.”

Female, 32, Ahero

The incentive facilitated drug adherence by helping buy food to manage the side effects of ARVs:

“*The drugs [ARVs] are powerful and without proper food*, *may cause someone to develop adverse side effects and eventually cause them to stop using them*.”

Male, 40, Rongo

### Limitations of economic incentives

We sought counter-evidence for the above observations, and in doing so identified circumstances and psychological dispositions of individuals for whom incentives may have had limited influence on care-seeking behaviors. Some participants were intrinsically motivated to seek care even in the absence of economic incentives. For these patients, the incentives merely ‘sweetened the deal’.

“*I used to come the clinic even when there was no incentive*, *but introduction of incentives is like when you introduce sugar into porridge that you previously took without sugar*.”

Female, 24, Lumumba

Importantly, the offer of incentives did not appear to “crowd out” or diminish intrinsic motivation for care engagement. A woman below discusses the importance of ARVs, and expressed that even if she used the incentive for transport, the incentive was not her only motivating factor for care-seeking:

“*My life depends on the drugs*, *not the incentive*. *Money is something that can come to pass […] what I am carrying in my bag is my life forever*. *It is the drugs*. *They asked me if I would not come if there is no transport… I told them that I would just come*, *because my life doesn’t depend on transport*, *it depends on something else*.”

Female, 22, Rongo

Participants’ intrinsic motivation to seek care was driven by desire to live a long, healthy life, to be able to care for children, and to achieve life aspirations. A woman below discusses the importance of her health and ability to take care of her children, as a motivating factor for care seeking, and further indicates she would continue to seek care even in the absence of economic incentives.

“*I will come whether there is transport [incentive] or not*. *I will just come to the clinic*. *So that I can take good care of my life and that of my children*.”

Female, 43, Lumumba

The participant below discusses adhering to treatment to attain childbearing and child rearing aspirations- highlighting the importance of family as an intrinsic motivator.

“*There is nothing wrong with the incentives […] once you have contracted HIV*, *you have to offer yourself to come for medication so that you may take care of your life and also for your loved ones*. *Like I have a child—I wish to add more children and that one isn’t enough for me*. *For me to be able to add more*, *I have to come for medication and take care of my life*, *so that I can be strong and later on I can add more children as I wish*.*”*

Female, 23, Ahero

Intrinsic motivation for care engagement was reinforced by witnessing other PLHIV succeed on care and treatment.

“*I have an aunty at home who has been on medication since the 90s and is still alive to date; her children are older than me and they are all educated*, *[…] she told me that I will be able to live even for the next 50 years if I continue taking medication*. *If she would have not been consistent with the medication*, *she would have died a long time ago and she wouldn’t have been able to educate her children*. *She would encourage me in that manner* …*”*

Female, 29, Migori

“*There are some two women from my area who lost their husband a long time ago only to later find out that they were HIV positive*. *They embraced using the drugs and are currently living very healthy lives even educating their children*. *That case has really encouraged me to continue coming to the clinic*.*”*

Male 27, Rongo

Similarly, witnessing negative examples reinforced care-seeking. Some patients reported seeing PLHIV that had failed to adhere to treatment and were themselves motivated to stay engaged in care to avoid the perceived negative consequences of ART non-adherence.

“*There is some lady from my area who died because of the disease—she began using medication then defaulted*. *I said that she was so foolish to stop taking medication yet the medication is provided for free*. *I vowed to continue taking the medication because it’s like my second God*. *Medication has got me far—I was very thin*, *but now I have added weight…Yes—my second God*, *because if I would quit the medication then I would have died already*, *and there wouldn’t be anyone to take care of my children*.*”*

Female, 29, Migori

Care engagement was reinforced by social support. For some, support from their spouses, peers, family members, and others, encouraged their care-seeking. While the incentives were appreciated and helpful, in instances where patients were otherwise strongly supported to seek care, they reported that incentives did not have a great deal of influence on their behavior.

“*I had to go and tell my wife first*. *This really helped me*, *since when I came back here from Rawaru*, *I came back with my wife and she was also tested—and so we always come for HIV treatment together*. *Even today I was with her*.”

Male, 30, Migori

“*[My husband] asks me who will take care of my child if I pass away*, *yet the child’s life is dependent on the mother*. *He told me to take that decision*. *Therefore*, *I would say that I decided to take the HIV medication because of him*.*”*

Female, 23, Ahero

“*At first it was challenging*. *You know when she [wife] went*, *she was immediately placed on drugs […] So I was wondering why I was only given Septrin*, *yet she came back home with a number of drugs*, *they were about 3 or 4 types […] I believed that even if I died then*, *there was nothing to lose*. *She reminded me that I had accepted my condition and so I should live positively*. *That really encouraged me and thus I have honored my clinic appointments to date*.”

Male, 30, Lumumba

“*The landlady asked me recently if I had gone to the hospital*, *since she was seeing that I am sickling*. *I told her that I had gone*. *She also asked if I had been started on care and I agreed*. *She then encouraged me to adhere to drugs stating that many people are on HIV medication*. *That made me encouraged to take medication*.”

Male, 37, Lumumba

Overall, participants who expressed a sense of optimism about the future, with fewer worries about dying because they were knowledgeable about the efficacy of ARTs. They had seen and experienced the impact and benefit of ARTs, and were strongly motivated to continue seeking care.

“*I will come because I’m the one who wants life; life is more important than work*. *If you are not alive*, *you can’t work*. *My HIV medication is my life right now and I give it top priority*.”

Female, 22, Migori

“*Everyone has his or her own challenges*. *For example*, *I have a sister*, *an in-law who was turned HIV positive even before I was born*. *She is still alive today and is very healthy because she adheres to his drugs*. *This is very encouraging for me—it gives me confidence that I can also live long*, *and motivates me to adhere to my HIV medication*.”

Female, 24, Lumumba

“*I can’t really say that the [incentive] makes me come to the clinic*. *It is a personal decision to come to the clinic and with or without the [incentive]*, *[…] coming to the clinic is compulsory to me*. *Even when I started coming to the clinic*, *I didn’t do it because there was some [incentive]*. *I came because I wanted to know how my health is doing*, *to get my HIV medication so that I can remain healthy*.”

Male, 40, Rongo

Finally, while the positive intrinsic and external motivators for care seeking appeared to moderate the influence of economic incentives on care-seeking, among some participants incentives were mistrusted as conspiratorial. For these patients, incentives were tainted and associated with perceived sinister motives and intentions of researchers, which limited their potential impact. Some expressed concerns about incentives being associated with “devil-worship” and nefarious activities. Participants below discussed prior experiences within their community, and narrated how they questioned the motives behind incentives being offered.

“*They may think that it is from the devil worshippers—nothing comes for free*. *Someone can perceive it negatively…Why someone would just give them free fare*. *Some people fear free giveaways because they may be required to pay back in some way*.”

Female, 24, Ahero

“*That’s why I concluded it was illuminati*. *Some pastor told us about some schoolgirl who also joined illuminati […] I asked myself where the money we were being given as [incentives] could possibly be coming from*. *I vowed to come back and ask about it*.”

Female, 29, Migori

## Discussion

The *ADAPT-R* trial sought to incentivize PLHIV to address potential barriers to care engagement they may face early in the HIV-care cascade, and identify heterogeneity in patients’ responses to these incentives based on their diverse needs. This qualitative study explored how small economic incentives facilitated patients’ ability to prioritize clinic attendance, following diagnosis of HIV and during the early stages of HIV care linkage, when the risk for economic instability and food insecurity are heightened [35,36]. We anticipated that incentives would alleviate the burden of paying for transport to clinic—well-documented as a major barrier to care engagement—and this key hypothesis underlying the trial design was confirmed in these qualitative findings. Numerous studies have documented the patient costs of HIV care in SSA, and the potential impact of small incentives in helping patients overcome costs incurred while accessing care, in the preliminary phase of linkage and initiation of ART [16,18,37]. Studies have also documented the role of incentives in influencing retention in care, which is critical to optimal HIV care-engagement [14–16,19,20,37]. With the introduction of incentives, patients in our study reported paying for costs of transport with the incentives, resulting in reduced time spent getting to and from clinics, less fatigue in walking long instances to access clinics, and a reduction time spent away from work.

Other findings were unanticipated and have enabled a deeper understanding of the pathways through which economic incentives influence HIV care-seeking behavior. What on the surface appeared to be a practical structural intervention (money) to alleviate a cost burden (transport to clinic) was revealed to lead to multi-dimensional consequences that facilitated care-seeking: having the ability to pay for clinic transport relieved individuals from the worries related to indebtedness to others, facilitated control over one’s schedule and ability to plan and prioritize, and expanded the range of available day-to-day life choices, enabling individuals to prioritize care-seeking as an act of self-care (attending clinic, adhering to medications, feeding oneself nutritious food). A spillover effect of this was an expanded ability to fulfill social role expectations as community members (confident of one’s ability to repay debts) and family members (happy to be able to buy and prepare household goods and food for spouses and children).

Patients reported paying outstanding debts and felt relief from the anxiety that they had previously experienced because of needing to borrow money for transport may thereby have potentially supported their ART adherence, as anxiety has been found to be strongly associated with non-adherence [38]. Patients also reported increased choice and empowerment, weighing the benefits of clinic attendance with the perceived gain of incentives, versus the costs of loss in productivity because of time away from work. Patients reported that incentives, in addition to helping address finances and transport barriers, enabled them to better address competing needs associated with clinic attendance and general HIV care, in alignment with previous findings [20]. In our study, incentives were reported to positively weigh these opportunity cost and benefit calculations, and helped prioritize clinic attendance.

The incentives were also reported to reduce food insecurity among households, in a setting where up to 80% of Kenyans have been documented to live under circumstances of poverty [39]. Food insecurity is a well-documented challenge, especially among household and individuals impacted by HIV [40]. Taking antiretroviral treatment without food can jeopardize health outcomes as a result of suboptimal response to ART by enhancing hunger or appetite, worsening ART related side effects and non-adherence to ART due to hunger [41]. The incentives were not only reported to have reduced food insecurity for patients, but were credited for the expansion of nutritional options, with patients reporting the purchase of nutrient-rich fruit, bread, vegetables, and meats. The family context in this setting, and in SSA in general, also underscores the interdependence of individual and family food and financial security [42]. Supporting this, patients highlighted pathways to food security that suggests that working and earning money to buy food and having money to get food to take with drugs was important not just for patients, but for their families, as has been shown in previous studies [43]. The sense of role fulfillment expressed by patients in being able not only provide for themselves but for their spouses and children was evident in our narratives.

In addition, our study found that incentives may play an inconsequential role for intrinsically motivated individuals. These individuals prioritized their ART regimens, clinic appointments, and were unwavering in their commitment to HIV care and their overall health. In these instances, the incentive may have served as a ‘carrot on top’ [44] and a helpful cue to action.

Finally, the incentive in this study was reported to fuel mistrust and evoke suspicion and negative attitudes among some community members. Participants questioned the source and motivation for the incentives, and associated it with ‘devil worship’. Research studies, especially in SSA and in HIV, are often met with mistrust, which may serve as a barrier to participant involvement [45]. Mistrust arises from conspiracies related the existence of HIV itself, funding sources, and suspicions of motives of the western world [46–49]. This can be mistrust of the communicators, the health care system, or the research information itself [48–50]; mistrust of funding sources in Africa has been associated with satanic rituals, especially when blood collection is involved [51,52]. Research mistrust and previous community experiences need to be addressed head on, prior to implementation, especially when studies involve money or incentives. Trusted local leaders and community members may be instrumental in dispelling myths and obtaining community trust [49].

This study was subject to limitations as data were gathered at baseline HIV care enrollment, and are reflective of an early phase in HIV care engagement. This study is also based on a sample of 39 individuals which may not be generalizable, and occurred in the context of rapidly shifting national guidelines for ART-for-all. However, this qualitative study includes plans for longitudinal data collection, which will help explore continued experiences with incentives, and their role in sustained care engagement and viral suppression. Further, we present rich qualitative findings from data gathered across varied clinic settings (urban/peri-urban), which contribute to the strength of our approach.

## Conclusions

Findings suggest that economic incentives may act to improve HIV care engagement via multidimensional pathways. By enabling an expansion of day-to-day life choices, incentives not only reduced transport barriers and food insecurity among patients and their families, but alleviated anxiety and helped PLHIV to prioritize care-seeking. Patients were able to borrow money for care-seeking, reported feeling relieved, less indebted, more autonomous, and better able to support others, which further enhanced their social role fulfillment. The ways in which the incentives were used suggests that having food, which includes earning money to buy food, and having money to buy food to take with ARVs, are important ways incentives function to improve outcomes for patients and their families in this setting. Thus, an important pathway of incentives to care engagement is via food security. Incentives had limited utility among intrinsically-motivated patients who already prioritized care-seeking.

## Supporting information

S1 Checklist COREQ checklist. (PDF)

S1 Table Selected in depth interview guide questions and probes. (DOCX)
