# Correction: Attitudes towards and experiences with economic incentives for engagement in HIV care and treatment: Qualitative insights from a randomized trial in Kenya

**DOI:** 10.1371/journal.pgph.0004868

**Published:** 2025-06-30

**Authors:** Sarah Iguna, Monica Getahun, Jayne Lewis-Kulzer, Gladys Odhiambo, Fridah Adhiambo, Lina Montoya, Maya L. Petersen, Elizabeth Bukusi, Thomas Odeny, Elvin Geng, Carol S. Camlin

The hyperlink in the Data Availability statement is incorrect. The correct hyperlink is: https://doi.org/10.6084/m9.figshare.28613414.
