# Supplementary material for: Attitudes towards and experiences with economic incentives for engagement in HIV care and treatment: Qualitative insights from a randomized trial in Kenya
Source: PLOS Glob Public Health. 2022 Feb 23;2(2):e0000204. doi: 10.1371/journal.pgph.0000204 (PMC10021832; doi:10.1371/journal.pgph.0000204)
Supplement: S1 Table — (DOCX) [file pgph.0000204.s002.docx]

**Supporting Documentation.**

**S1 Table. Selected In-depth Interview Guide Questions and Probes.**

| **Thematic Area** | **In-depth Interview Questions (Illustrative)** |
| --- | --- |
| **Perceptions and attitudes related to HIV care engagement** | [If respondent was previously enrolled in HIV care, but dropped out prior to start of ADAPT study:]   - Please tell me about the events that led up to your dropping out of care. What was happening in your life, or at the clinic, that made it hard for you to stay in care?   [If respondent newly enrolled in HIV care:]   - What things in your life help you to make your clinic appointments? *Probe for things that respondent does or that other people in his/her life do that help him/her to stay in care.* |
| **Reactions / emotional responses to learning about transport voucher** | Let’s take a minute for you to recall the day our study team member told you about the transport voucher.   - Can you remember that day? Where were you? What were you doing at the time? - What did the study team member tell you about the transport voucher? And what was your first reaction to learning about the transport voucher? - And what else were you thinking, about the transport voucher? - How did it make you feel to hear about it? |
| **Discussions/sharing about transport voucher** | - Who did you tell about the transport voucher you were offered? And how did they respond? - Were there people you wanted to tell, but felt you couldn’t? Please tell me about that. |
| **Perceived influence of incentive on care decision/care seeking behaviors** | Let me ask you some more about that transport voucher, and about your decision whether to come or not to come to [clinic name] to seek HIV care and treatment.   - How did it affect your decision making about whether or not to come to this clinic to get treatment for HIV? |
| **Relationship, family and other contextual factors facilitative of HIV care-seeking** | I want to ask you about how other people in your community feel about getting treatment for HIV, especially your friends or family close to you.   - How do you think most of them feel about getting care and treatment for HIV? - To what extent do you and your friends talk about HIV treatment? What kinds of things do your friends say about it? - What are the main motivations for people to enroll in HIV care programs? And what about staying enrolled in HIV care? - Please tell me, what do you think are the main reasons why people do not seek care and treatment for HIV?   I want to ask you more about your main intimate and family relationships, and about how you and your partners have handled discussions around getting treatment for HIV.   - First, please tell me about your marital status, and about any girlfriends/boyfriends. Probe for whether respondent has a husband/wife or main partner with whom s/he resides, and any other sexual partners s/he sees regularly. - [If currently married] Is HIV testing something you and your spouse have talked about? Please tell me about that. - What are the things that make it hard to discuss HIV treatment with him/her? - What do you think might make it easier |
